# Managing Wound Complications After Osteosarcoma Resection: Stopping Adjuvant Therapy and Performing Secondary Closure

**DOI:** 10.7759/cureus.74365

**Published:** 2024-11-24

**Authors:** Kiichi Furuse, Daisuke Kageyama, Masaki Arikawa, Satoshi Akazawa, Takuya Higashino

**Affiliations:** 1 Plastic and Reconstructive Surgery, National Cancer Center Hospital East, Chiba, JPN; 2 Plastic and Reconstructive Surgery, National Cancer Center Hospital, Chuo City, JPN

**Keywords:** adjuvant chemotherapy, debridement, healing, limb salvage, osteosarcoma, retrospective studies, tumour, wound healing

## Abstract

Purpose

Adjuvant chemotherapy (AC) following limb-sparing surgery with endoprosthesis is the gold standard treatment for osteosarcoma (OS). However, AC can impair wound healing, leading to endoprosthesis exposure, making the decision to continue or pause AC important. We propose standard guidelines for managing this situation.

Methods* *

This observational retrospective study analyzed local findings, AC courses, wound complications, and overall survival of 22 patients who underwent resection of primary OS.

Results

Of nine patients with wound complications (41%), two achieved secondary healing before starting AC while the other seven patients had wound deterioration during AC. Six patients had temporary suspension of AC, followed by debridement and secondary closure, and the completion of AC, one had temporary suspension of AC with conservative therapy, but could not complete AC due to too long suspension of AC. No recurrence or metastasis was recorded. Comparing these nine patients with the other 13 patients without wound complications, the number of days from the operation to the end of AC was 150 days and 144 days respectively, and no statistical differences were observed (p=0.648).

Conclusion

Managing wound complications after OS resection requires balancing the completion of AC with effective limb salvage strategies*. *Deciding on temporary suspension of AC without delay and secondary closure is important.

## Introduction

Osteosarcoma is a primary malignant bone tumor that often originates from the metaphysis of the distal femur and proximal humerus; its incidence among malignant tumors is 0.20% [1]. Adolescents and young adults are at high risk of osteosarcoma; therefore, limb salvage is important when limiting the spread of the disease. Adjuvant chemotherapy (AC) and limb-sparing surgery with endoprosthesis are the gold standards of therapy, and the long-term survival rate of patients with osteosarcoma has increased to almost 70% with these treatments [2,3].

AC is essential to prevent recurrence and improve prognosis but sometimes deteriorates wound healing, which leads to endoprosthesis exposure and amputation. Chemotherapeutic agents delay DNA, RNA, or protein synthesis, resulting in reduced fibroplasia and neovascularization of wounds. In addition, these drugs inhibit cell migration into the wound, lower early wound matrix formation, impair collagen production, decrease the proliferation of fibroblasts, and inhibit the contraction of wounds [4]. For example, wound healing disorder due to chemotherapeutic agents such as doxorubicin is most common when the agents are administered preoperatively or within three weeks postoperatively [5].

Thus, deciding whether to continue AC for a better prognosis or postpone it to facilitate limb salvage is crucial in cases of wound complications after osteosarcoma resection. If AC is to be temporarily suspended, clinicians must choose between secondary healing and closure. However, no report has described the criteria on which this decision should be based. This study offers standard guidelines for managing this situation.

## Materials and methods

This was an observational retrospective study conducted at National Cancer Center Hospital, Japan. The Ethics Committee of the National Cancer Center, Japan, approved the study (approval number: 2018-158). This study was conducted following the principles of the Helsinki Declaration.

Patients who underwent resection of primary osteosarcomas of the proximal tibia or distal femur and reconstruction with modular knee endoprostheses and primary gastrocnemius flaps between January 2012 and December 2022 were included. Patients without pre- or postoperative chemotherapy were excluded. A total of 22 patients who fulfilled the inclusion and exclusion criteria were included in the study.

A tumor biopsy was performed to confirm the diagnosis before primary oncological resection. All patients had undergone chemotherapy pre- and postoperatively and had not received radiation therapy. Five patients without AC and one patient without primary gastrocnemius flaps were excluded. Local findings, AC courses, and overall survival were recorded. Wound complications were classified as deep infection, delayed wound healing, and partial necrosis of the skin graft. Deep infection was defined as a periprosthetic infection that met at least one of the following criteria: (i) purulent drainage from a surgical drain, (ii) purulent material in a joint, and (iii) positive growth of organisms in an aseptically obtained swab/tissue from the joint/bone [3]. Delayed wound healing and partial necrosis of the skin graft were diagnosed by inspection. Delayed wound healing included wound dehiscence and stitch abscesses.

The 22 patients comprised 12 men and 10 women aged 6-38 years (mean, 13.7 years; median, 13.5 years). Their cancer stages were IIA (seven patients), IIB (12 patients), III (two patients), and Ⅳa (one patient). The tumor sites included 13 distal femurs and nine proximal tibias. Among the 22 patients, 19 had high-grade osteosarcoma, one had low-grade central osteosarcoma with focal high-grade progression, one had pleomorphic sarcoma most consistent with osteosarcoma, and one spindle cell sarcoma with myogenic differentiation that was clinically treated as high-grade osteosarcoma. All diagnoses were histologically confirmed. Fifteen patients underwent direct closure, seven underwent slit-thickness skin grafting, and no cases of total necrosis of the skin graft occurred. Regarding the types of prostheses, the Kotz Growing prosthesis (Stryker Corporation, Kalamazoo, Michigan, United States) was used in six cases, Global Modular Replacement System® (GMRS) (Stryker Corporation) in six cases, Compress® (Zimmer Biomet Holdings, Inc., Warsaw, Indiana, United States) in five cases, KMLS® (Kyocera Modular Limb Salvage) System (KYOCERA Corporation, Kyoto, Japan) in one case, and a ceramic spacer in four cases. Surgical drains were removed when the output was < 20 mL per day or within 21 days postoperatively. AC was initiated as soon as all surgical drains were removed. For chemotherapy, doxorubicin, cisplatin, and methotrexate were administered according to the methotrexate, doxorubicin, and cisplatin (MAP) therapy protocol [6,7]. Ifosfamide was added to some of the standard responders postoperatively according to the JCOG0905 study [8]. The response rate of chemotherapy ranged from 10% to 100% (mean, 69%; median, 70%). There was no oncological difference between patients who had complete necrosis with neoadjuvant chemotherapy versus poor responders. The minimum follow-up period was one year (median, five years; range, 1-9 years). None of the patients were lost to follow-up. At the last follow-up, 19 of the 22 patients (86.4%) were alive without local recurrence or metastases, two patients were alive after ablation of lung metastases, and one patient died owing to lung metastases.

Data analysis

The Welch test was used for continuous variables to compare the differences between the groups with and without wound complications. Statistical significance was set at p < 0.05. All statistical analyses were performed using R software (version 4.3.1; R Foundation for Statistical Computing, Vienna, Austria).

## Results

Wound complications were observed in nine patients (41%) (Table 1), consisting of one patient with partial skin necrosis (case 1), six patients with delayed wound healing (cases 2-7), and two patients with deep infection (cases 8, 9). Two patients (cases 1, 4) achieved secondary healing before starting the AC treatment.

**Table 1:** The demographic characteristics of patients with wound complications NAC, neoadjuvant chemotherapy; AC, adjuvant chemotherapy; SG, skin graft; A, doxorubicin; P, cisplatin; AP, doxorubicin-cisplatin; #, number of courses; M, methotrexate; IFO, ifosfamide

Case	Age	sex	NAC	Complications	AC
1	14	F	AP#2, M#5	Partial SG necrosis	AP#2, M#7, A
2	7	M	AP#2, M#4	Stitch abscess	AP#2, M#6, A#2
3	7	M	AP#3, M#6	Dehiscence	AP, M#5, A#2
4	27	M	AP#2, M#5	Dehiscence	AP#2, M#6, A#2
5	9	F	AP#3, M#4	Dehiscence	AP, M#6, A#2
6	12	F	AP#3, M#6	Dehiscence	AP#1, M#2, A
7	13	F	AP#2, M#3	Dehiscence	AP#2, M#6, A#2
8	14	F	AP#2, M#4	Deep infection	AP, IFO#5
9	16	F	AP#2, M#4	Deep infection	M#8, AP#2, A#2

Deterioration of wound complications during AC

The postoperative courses of the patients with wound complications are shown in Table 2. Of the seven patients with wound complications during AC (cases 2, 3, 5-9), all the patients had wound deterioration. Six (cases 2, 3, 5, 7-9) underwent debridement and secondary closure and completed the AC course. The other patient (case 6) did not undergo secondary closure because we expected secondary healing with continuous AC, which was not achieved eventually.

**Table 2:** The postoperative day (POD) of each event AC, adjuvant chemotherapy; A, doxorubicin; P, cisplatin; AP, doxorubicin-cisplatin; #, number of courses; M, methotrexate; IFO, ifosfamide

Case	AC initiation	Finding complications	AC suspension	Debridement and secondary closure	AC resumption
1	POD 32	POD 9	Not necessary	Secondary healing before AC	Not necessary
2	POD 21	POD 46 (after AP)	POD 89 (after AP, M#4, A)	POD 93	POD 96
3	POD 21	POD 29 (after AP)	POD 29 (after AP)	POD 37 (after AP)	POD 49
4	POD 35	POD 10	Not necessary	Secondary healing before AC	Not necessary
5	POD 19	POD 13	POD 21 (after AP)	POD 43 (after AP)	POD 52
6	POD 22	POD 45 (after AP)	POD 68 (after AP#1, M#2, A#1)	None	Impossible
7	POD 19	POD 95 (after AP, M#4, A)	POD 140 (after AP#2, M#6, A#2)	POD 168	Not necessary
8	POD 20	POD 460 (after AP, IFO#5)	Not necessary	POD 463	Not necessary
9	POD 47	POD 11	POD 54 (after M#2)	POD 27, 69	POD 90

Postoperative course after secondary closure

No cases of delayed wound healing recurrence occurred after AC was restarted among the secondary closure cases in our study. In case 9, debridement was performed twice because the deep infection did not improve after the first debridement, and the modular prosthesis was exchanged with a ceramic spacer in the second debridement.

Course of wound complications with temporary suspension of AC and conservative therapy

**Figure 1:**
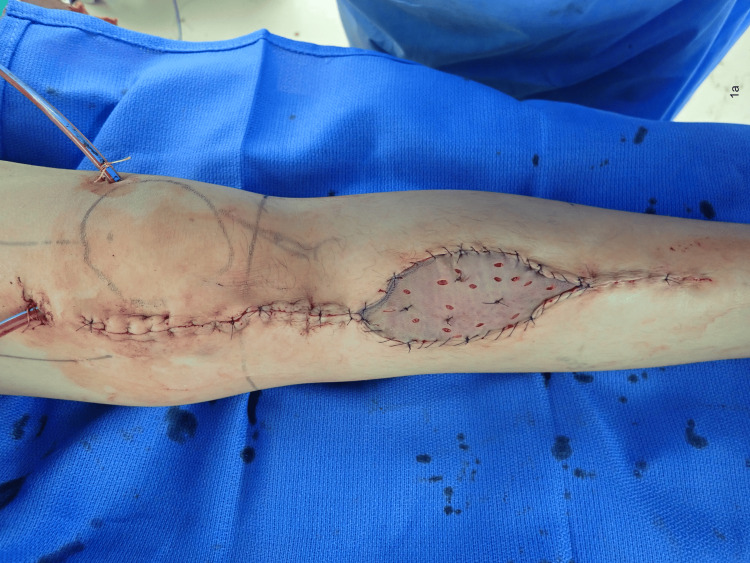
The surgical site immediately after tumor resection and reconstruction with prostheses, gastrocnemius flap, and skin graft

**Figure 2:**
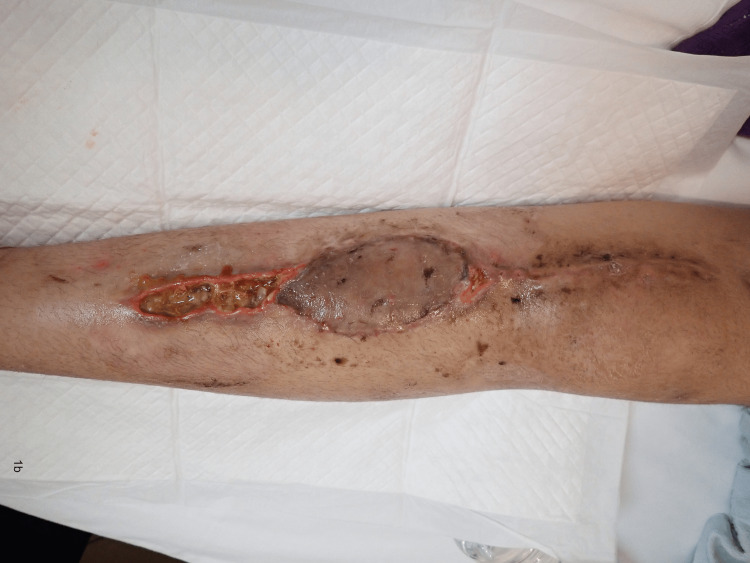
The surgical site on postoperative day 45 after adjuvant chemotherapy of AP (doxorubicin-cisplatin).

**Figure 3:**
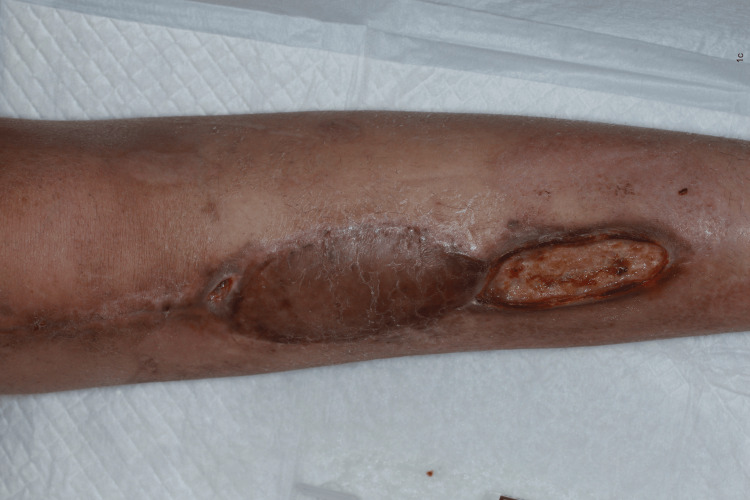
The surgical site on postoperative day 68 after adjuvant chemotherapy of APMMA APMMA:  One course of doxorubicin-cisplatin (AP), two courses of methotrexate (M), and one course of doxorubicin (A)

**Figure 4:**
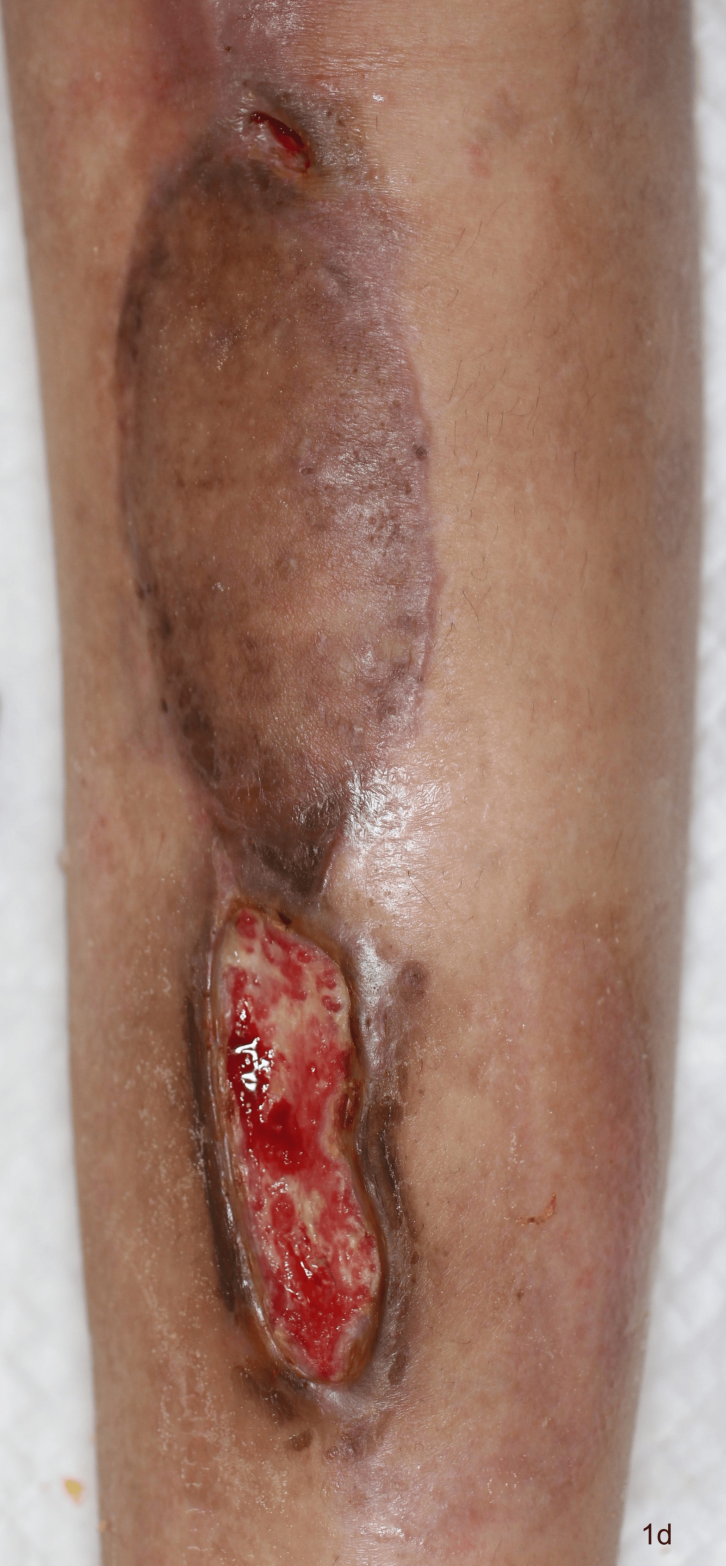
The surgical site on postoperative day 96 (28 days after the suspension of adjuvant chemotherapy)

**Figure 5:**
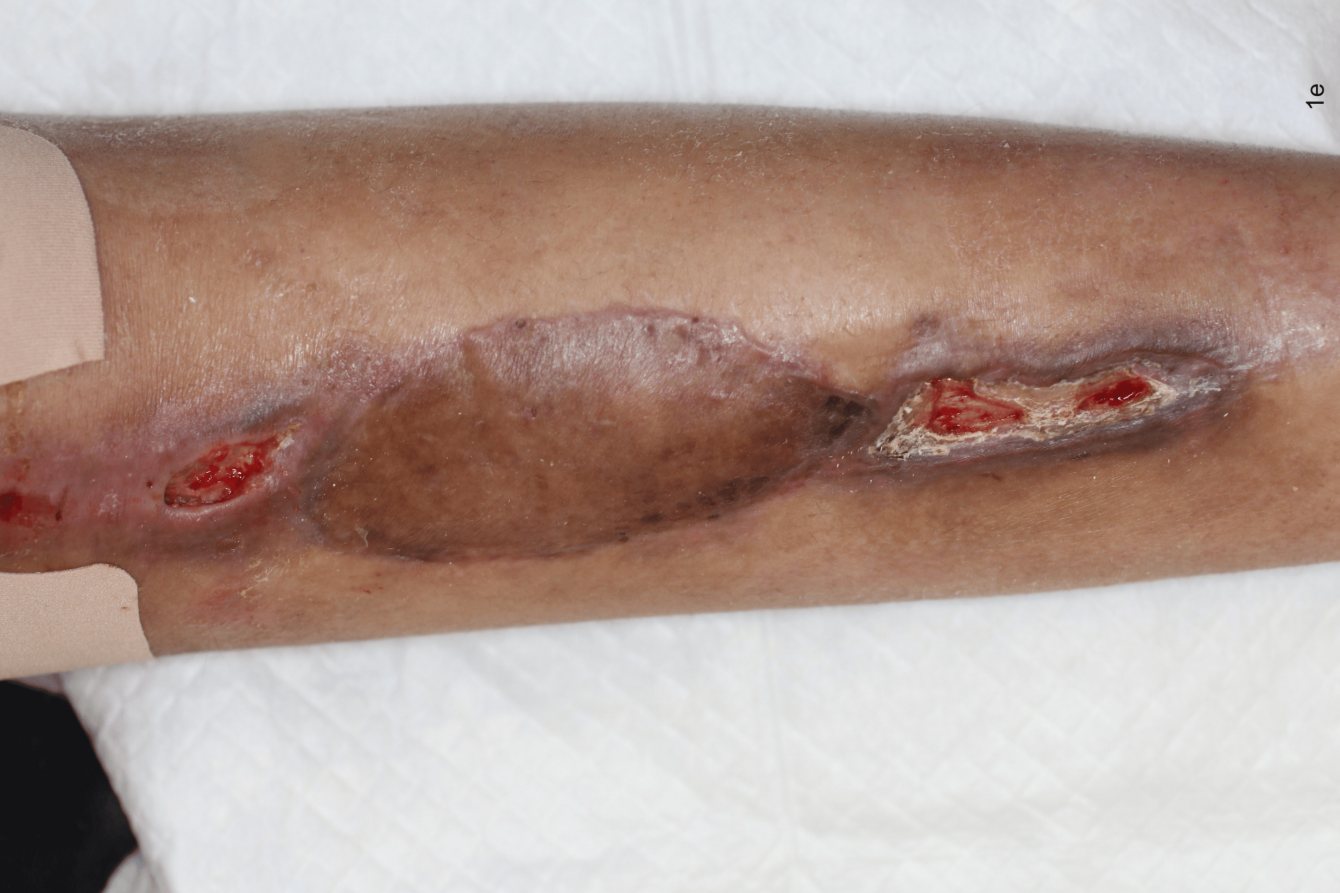
The surgical site postoperative day 110 (42 days after the suspension of adjuvant chemotherapy)

In case 6, we could not complete the AC course because chemotherapy was paused for three months to allow for secondary healing and prevent implant exposure. Figures 1-5 illustrate the local findings for case 6.

Oncological outcome

The nine patients with wound complications survived without local recurrence or metastases. No statistical differences were observed between these patients and the other 13 patients without wound complications in terms of the number of days from the operation to the initiation of AC, from the initiation to the end of AC, or from the operation to the end of AC (Table 3).

**Table 3:** A comparison between patients with and without wound complications

	Patients without wound complications	Patients with wound complications	P
Number of patients	13	9	
Mean follow-up period	4 years	4 years	
Number of AC dropouts	0	1	
Metastases	2	0	
Number of deaths	1	0	
Days from the operation to the initiation of AC	25.0	26.2	0.749
Days from initiation to the end of AC	119.4	124.0	0.693
Days from the operation to the end of AC	144.4	150.2	0.648

## Discussion

Limb-sparing surgery is currently the standard of care for primary bone tumors, especially in the pediatric population [9-12]. Complete remission of malignancies and uneventful wound healing are two fundamental factors for successful limb-sparing surgery; however, they are often contradictory.

First, although wide en bloc resection of bone and adjacent soft tissue is crucial for the complete resection of tumors, it interferes with wound healing owing to the large dead space and limited vascularity of the wounds. The surgical risks of oncologic total knee replacement are well-defined for infections as approximately 10-15% with additional soft tissue or wound healing issues of 15-20%, which is much higher than that for conventional arthroplasty [13-18]. We addressed this problem by transposing well-vascularized tissues to provide stable prosthetic coverage. The gastrocnemius is a well-suited flap owing to its large muscle belly, robust vascular supply, and shorter arc of rotation around the knee [13,19]. Furthermore, its transfer does not negatively affect extremity function.

Second, AC greatly prevents local recurrence and metastasis of osteosarcomas in the pediatric population, although it affects wound healing. Additionally, many treatments promoting wound healing (including basic fibroblast growth factor agent spraying and negative-pressure wound therapy) are contraindicated after tumor ablation in general. Reports discussing ways to strike a balance between complete remission of osteosarcoma and uneventful wound healing are lacking.

**Figure 6:**
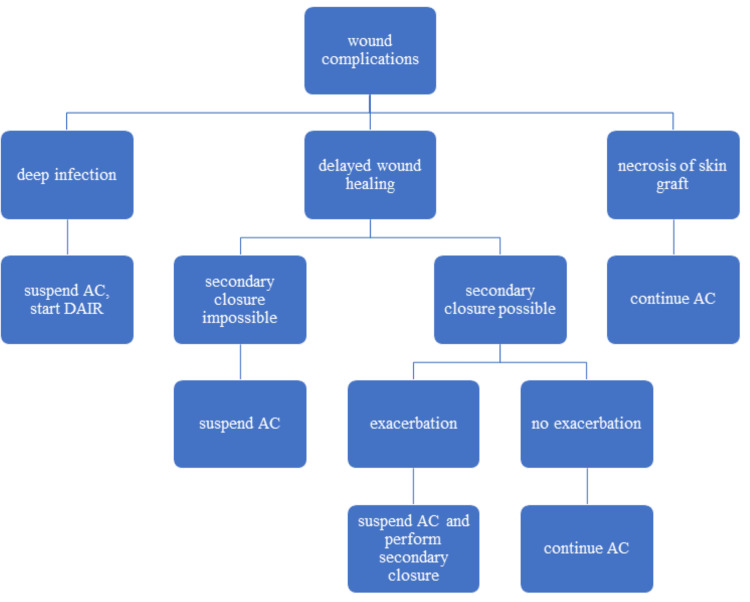
Proposed protocol for wound management AC, adjuvant chemotherapy; DAIR, debridement, antibiotics, irrigation, and implant retention

Our proposed wound management protocol is shown in Figure 6.

If the wound complication is a deep infection, AC should be temporarily suspended, and DAIR (debridement, antibiotics, irrigation, and implant retention) should be performed. Although the optimal management for early and acute periprosthetic joint infection of lower extremity reconstructions is still debated, a DAIR procedure with the exchange of all modular components (DAIR plus) has been reported to result in satisfactory infection control [20]. AC can be restarted if the patient’s general condition (including body temperature and laboratory data) permits.

If the wound complication is delayed wound healing, this becomes problematic because it rarely affects systemic conditions or lab data, and the clinician must decide whether to continue or suspend AC. Our case series suggests that AC continuation is acceptable only when wound dehiscence is sufficiently small for secondary closure and is not exacerbated. Once wound dehiscence begins to increase, secondary closure should be planned before it becomes impossible.

The decision to perform secondary closure is often difficult because it requires AC suspension and general anesthesia in the pediatric population; however, it eventually minimizes the period of AC suspension. All patients who underwent debridement and secondary closure completed the AC course, and no statistical difference existed in the number of days of chemotherapy compared with those who did not have wound complications. This may be because there are several reasons for the temporary suspension of AC other than wound complications, including neutropenia and upper respiratory tract infections.

No cases of delayed wound healing recurrence occurred after AC was restarted among the secondary closure cases in our study. This may be attributed to a better wound environment during the secondary closure than the first operation with tumor ablation. In case 6, we could not complete the course of AC because secondary healing took longer than expected. According to our proposed protocol, we should have suspended the AC in case 6 at the time illustrated in Figure 2 because the wound dehiscence already seemed too large for secondary closure.

All cases of delayed wound healing eventually required secondary closure, except in case 4, wherein wound dehiscence improved before AC was started. This suggests that secondary healing with continuous AC is not the treatment of choice, although it seems reasonable. This is also assumed to be true when comparing Figures 2, 3.

In a study by Imran et al., a delay of over 21 days in resuming chemotherapy after definitive surgery was associated with an increased risk of death in patients with localized osteosarcoma in an extremity [21]. In our study, the median number of days in the resumption of chemotherapy was 22.5 days, and only one patient (4.5%) died. Three reasons may explain this discrepancy. First, 116 patients (23.2%) had amputation in the previous study [21] with shortened days in the resumption of chemotherapy. In our study, no patients had to undergo amputation. Second, unlike our series, Imran et al. did not state whether each patient received all of the planned chemotherapy cycles or if other delays existed between chemotherapy courses, including a delay in the performance of definitive surgery, either of which may have underestimated the true days necessary for chemotherapy resumption [21]. Finally, our series may have been too small, and the follow-up periods are too short to conclude.

Our study is limited by its retrospective nature and the limited number of cases owing to the rarity of osteosarcoma. We cannot answer our questions from this small heterogeneous series with a relatively short follow-up and a mix of femoral and tibial sites, which have differing likelihoods of wound complications. Nevertheless, our result deserves attention because we propose a guideline that further studies can follow and check.

## Conclusions

Managing wound complications after osteosarcoma resection is important in striking a balance between the completion of AC and limb salvage. Secondary healing with continuous AC is not a reasonable treatment of choice. If possible, a decision should be made about the temporary suspension of AC without delays and secondary closure. The postoperative course of secondary closure is generally uneventful, and the temporary suspension of AC does not significantly extend the total period of AC.
